# Global Patent Landscape and Technological Trends in Biosafety Level 3 (BSL-3) Laboratories Technologies

**DOI:** 10.3390/biotech15030052

**Published:** 2026-07-10

**Authors:** Milca de J. Silva, Roni D. Vinhas, Helena S. da Hora, Saada L. C. Fernandez, Hayna Malta-Santos, Hugo Saba, Camila D. F. Ribeiro, Marilda de S. Gonçalves, Bruna A. S. Machado

**Affiliations:** 1 SENAI Institute of Innovation (ISI) in Health Advanced Systems (CIMATEC ISI SAS), SENAI CIMATEC University, SENAI CIMATEC, Salvador 41650-010, BA, Brazil; milca.jesus@fieb.org.br (M.d.J.S.); helena.hora@fieb.org.br (H.S.d.H.); hayna.santos@fbter.org.br (H.M.-S.); 2 Postgraduate Program in Industrial Management and Technology, SENAI CIMATEC University, SENAI CIMATEC, Salvador 41650-010, BA, Brazil; roni.vinhas@fiocruz.br; 3 Gonçalo Moniz Institute, Oswaldo Cruz Foundation—Fiocruz, Salvador 40296-710, BA, Brazil; marilda.goncalves@fiocruz.br; 4 Oswaldo Cruz Foundation—Fiocruz, Rio de Janeiro 21040-900, RJ, Brazil; saada.fernandez@fiocruz.br; 5 Department of Exact and Earth Sciences, State University, Salvador 41150-000, BA, Brazil; hugosaba@gmail.com; 6 Nutrition School, Federal University of Bahia, Salvador 40110-907, BA, Brazil; camiladuartef@ufba.br; 7 Graduate Program in Food Science, Faculty of Pharmacy, Federal University of Bahia, Salvador 40170-290, BA, Brazil

**Keywords:** BSL-3 laboratories, patent analysis, biosafety technologies

## Abstract

Biosafety Level 3 (BSL-3) laboratories are essential for handling high-risk pathogens and strengthening global health security. This study presents a patent landscape analysis of BSL-3-related technologies using the Derwent World Patents Index (DWPI) to identify technological trends, geographic distribution, patent classifications, and temporal evolution. Patent documents associated with laboratory infrastructure, ventilation systems, containment devices, and biosafety procedures were screened and analyzed. A total of 58 patent documents filed between 2009 and 2024 met the inclusion criteria. The results showed that China and the United States are the leading contributors to BSL-3 patent development, reflecting continued investments in biosafety and biosecurity infrastructure. The most frequent International Patent Classification (IPC) categories were C12M (microbiological devices), E04H (specialized construction infrastructure), and F24F (ventilation and air control systems), highlighting the multidisciplinary nature of innovations in laboratory containment and safety. The temporal trends revealed increases in patent activity following major public health emergencies, including SARS, Ebola, and particularly the COVID-19 pandemic. Furthermore, a significant increase in patent expirations is expected by 2029, creating opportunities for technology transfer, open innovation, and broader access to critical biosafety technologies. These findings emphasize the strategic importance of continued investment in BSL-3 technologies, especially in developing countries with growing biosafety demands.

## 1. Introduction

In recent decades, advances in biotechnology have increased the need for biosafety and biosecurity regulations [1] due to the risks associated with handling biological agents and the potential misuse of pathogens [2]. The emergence of infectious diseases such as COVID-19, avian influenza, Middle East respiratory syndrome (MERS), and Ebola has reinforced the importance of effective risk management and quality assurance systems for global health protection [3]. This landscape demands not only a reactive response, but the implementation of robust risk management and quality assurance systems to ensure proactive prevention and operational excellence.

In this context, biosafety plays a central role in the development and implementation of measures for the safe handling of biological materials. It aims to protect human, animal, and environmental health through the implementation of regulations, technical procedures, and preventive strategies related to exposure to pathogenic organisms and emerging diseases [4]. In addition, biosecurity encompasses a set of measures designed to respond to intentional biological threats—such as the malicious use of biological agents (viruses, bacteria, or toxins) for the purposes of sabotage, bioterrorism, or biological warfare—and to restrict access to sensitive materials and facilities. This strengthens institutional security and ensures the protection of strategic laboratory processes [5].

Biosafety encompasses several complementary dimensions, including the safe handling of pathogenic microorganisms, operational biosafety practices, laboratory infrastructure and facility management, and preparedness for emerging biological risks [5]. Together, these components provide the foundation for preventing laboratory-acquired infections and ensuring the safe operation of high-containment laboratories [6]. Among these dimensions, technological innovation plays a critical role in strengthening BSL-3 laboratory infrastructure and operational capacity.

In this scenario, high-containment laboratories, particularly those classified as Biosafety Level 3 (BSL-3) or Level 4 (BSL-4), are essential for the research, diagnosis, and handling of highly pathogenic microorganisms [6]. Their integration strengthens the emergency response capacity and international scientific cooperation against biological threats [7].

BSL-3 laboratories are used in clinical, diagnostic, research, or production activities involving biological agents capable of causing serious or lethal diseases through inhalation. These represent significant risks to both laboratory personnel and the environment [8]. These facilities require specialized training, continuous supervision, and biocontainment systems such as HEPA filtration and controlled airflow to prevent pathogen release. From a quality management perspective, the operational efficiency of a BSL-3 laboratory includes airflow control, waste management, and equipment maintenance, all of which are essential to ensuring safety and scientific reliability [9].

BSL-4, on the other hand, represents the highest level of biological containment and is reserved for work with microorganisms that present high individual and community risks, with the potential to cause severe or fatal diseases, transmitted via aerosols or whose transmission routes are not yet fully understood [10]. Access to these laboratories is strictly controlled and limited to highly trained professionals who operate using pressurized suits and life-support systems designed to ensure maximum protection [11]. Additionally, these facilities rely on multiple containment barriers and standardized operating procedures that guarantee the absolute confinement of the agents being handled [12].

Due to the high costs of construction, operation, and personnel training, BSL-4 laboratories require stricter protocols, frequent inspections, and highly specialized infrastructure, limiting their global distribution. They are generally located in physically isolated facilities, disconnected from other laboratory units [13,14]. In contrast, BSL-3 laboratories are more technically and economically feasible, leading to wider implementation worldwide [15]. Consequently, investing in the continuous optimization of BSL-3 processes through technological innovation is not only a safety issue but also one of operational sustainability. Although they operate at lower containment levels, BSL-3 facilities still ensure the safe handling of high-risk pathogens and require rigorous biosafety standards. Their lower costs, flexibility, and accessibility make them the preferred choice for many governments and research institutions [16].

Within this context, there is a growing interest in technological innovations related to advancements in Biosafety Level 3 (BSL-3) laboratories. Investments in this area have attracted attention from various sectors, especially on patents related to the construction, operation, and improvement of such facilities, as well as the development of products and systems applicable to the BSL-3 environment [17].

A patent analysis is a strategic tool for understanding investment trends and identifying emerging technologies in this field [18]. Such an analysis also supports benchmarking, competitive intelligence, and the identification of opportunities for standardization, automation, and operational efficiency. Furthermore, technological mapping can guide public and private investments, strengthen biosafety infrastructure, and improve the response capacity to biological threats.

## 2. Materials and Methods

A methodological approach combining qualitative and quantitative analyses was employed to investigate technologies related to Biosafety Level 3 (BSL-3) laboratories. Patent data were retrieved from the Derwent World Patents Index (DWPI, Clarivate Plc, London, UK) database, available through Clarivate Analytics and accessed via SENAI CIMATEC’s institutional license.

For patent document selection, a search strategy was applied using the DWPI Advanced Search module, combining keywords related to technologies, equipment, systems, and infrastructure associated with Biosafety Level 3 (BSL-3) laboratories. The search terms were combined with wildcards (*), quotation marks (“ ”), and Boolean operators (OR/AND). Wildcards were used to capture morphological variations of terms (e.g., singular and plural forms), quotation marks ensured the retrieval of exact expressions, and Boolean operators enabled the identification of documents containing at least one relevant descriptor (OR) or all descriptors simultaneously (AND).

In addition, advanced search commands targeting patent titles, abstracts, claims, and International Patent Classification (IPC) codes were employed. The search terms were defined based on controlled vocabulary and technical descriptors related to biosafety, containment, high-risk pathogens, and laboratory infrastructure. The following Boolean expression was applied to ensure the comprehensive retrieval of relevant patent documents:

(Instrument* OR Equipment* OR Structure* OR Apparatus* OR Tool*) AND (Biosafety) AND (“BSL-3” OR “Biosafety Level 3” OR “High-Containment Laboratory”).

The search strategy combined broad technological descriptors (“instruments”, “equipment”, “structures”, “apparatus”, and “tools”) with biosafety-related terminology and specific references to BSL-3 and high-containment laboratory environments. This approach was designed to maximize the retrieval of patent documents explicitly associated with the operational and technological context of BSL-3 laboratories.

The search conducted in 2025 retrieved 258 patent documents from the DWPI, corresponding to patent applications filed between 2009 and 2024. As a first screening step, only active patents were selected, resulting in a dataset of 156 patent documents. Subsequently, a manual review was performed to exclude duplicate records, operational procedures, and documents not directly related to the scope of BSL-3 laboratory technologies. This screening process resulted in a final dataset of 58 patent documents for a detailed analysis. The bibliometric and technological indicators provided by the DWPI and selected for the analysis included the publication year, estimated patent expiration year, country of origin, applicants, and inventors. The estimated patent expiration year was obtained directly from the DWPI, which provides this information for each patent record. All data were available in the database, and no additional calculations, projections, or estimations were performed by the authors. Screening and categorization were performed using Microsoft Excel spreadsheets, enabling the efficient organization and tracking of the selected documents. Figures and diagrams were created using BioRender (BioRender Inc., Toronto, ON, Canada), Canva (Canva Pty Ltd., Sydney, NSW, Australia), and Flourish (Flourish Studio Ltd., London, UK), while statistical graphs were generated using GraphPad Prism 8.0 (GraphPad Software LLC, San Diego, CA, USA). No artificial intelligence (AI)-assisted tools were used during the preparation of this manuscript. All stages of the study, including the literature review, study design, data collection, data analysis, interpretation of results, manuscript writing, and revision, were conducted exclusively by the authors.

## 3. Results and Discussion

### 3.1. Patenting of BSL-3 Technologies Accelerated After 2015 and Reveals New Opportunities for Future Innovation

**Figure 1:**
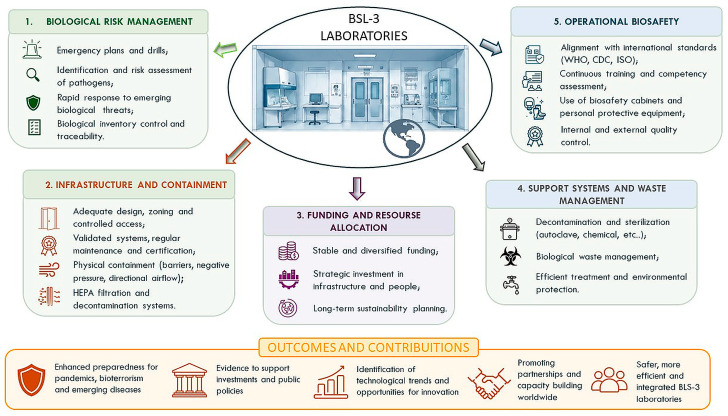
**Conceptual framework of the study.** The scheme illustrating the relationships among biosafety principles, BSL-3 laboratory technologies, funding and infrastructure requirements, operational biosafety, support systems, and the expected contributions of technological innovation to global health security. The framework also highlights the role of patent landscape analysis in identifying technological trends and innovation opportunities. The arrows indicate the conceptual relationships between BSL-3 laboratories and each major component, whereas the different colors are used solely to distinguish the thematic domains represented in the framework.

The development of BSL-3 laboratory technologies has a long-standing history. The pandemics that humanity has experienced over time have undoubtedly contributed to increased efforts to build robust and safe facilities, as well as to investment and R&D in this sector in China [19]. To provide a broader context for the technological landscape analyzed in this study, Figure 1 presents a conceptual overview of the key components that support BSL-3 laboratories, including biological risk management, infrastructure and containment systems, funding and resource allocation, operational biosafety, and waste management. The framework also highlights how technological innovation in these areas contributes to strengthening biosafety infrastructure and global health security.

**Figure 2:**
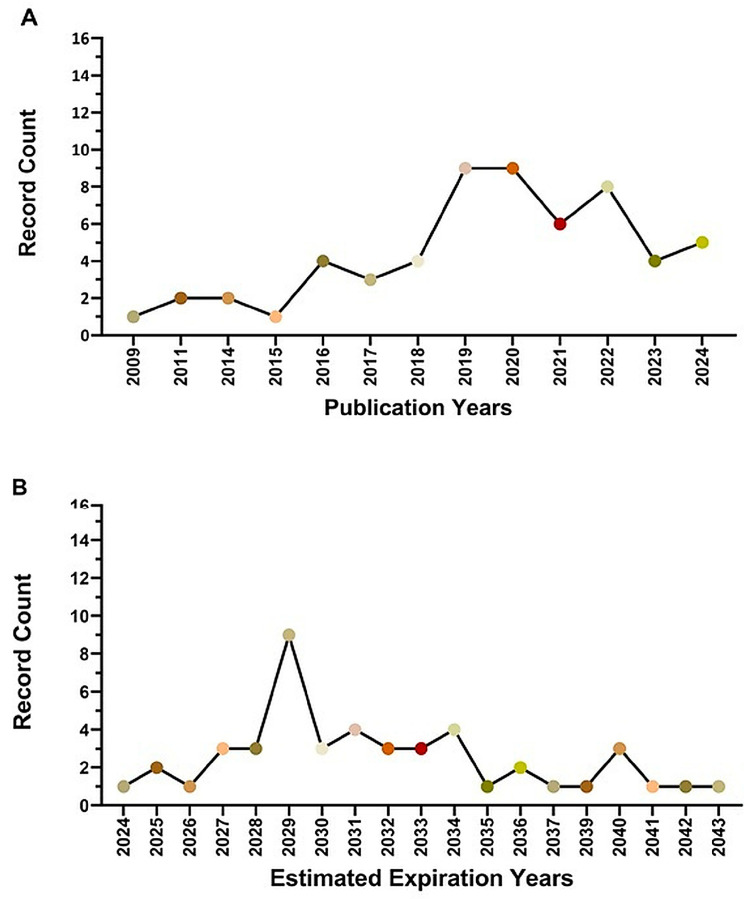
**Annual patent distribution profile.** (**A**) Patent filings related to BSL-3 technologies from 2009 to 2024. (**B**) Projected expiration trends of BSL-3 patents. The colored markers are used solely to improve visual differentiation between consecutive data points and do not represent different patent categories or technological classifications.

In this article, a patent search was conducted to assess the current state of the technology, equipment, systems, and infrastructure related to BSL-3 laboratories. An analysis of trends in patent applications in this field up to 2015 showed that research and innovation in this area still accounted for a small number of applications. Since then, there has been an overall increase, with a peak in applications in 2019–2020 (Figure 2A).

It is important to note that the Chinese Academy of Military Medical Sciences has allocated significant resources to this area, launching major infrastructure development projects as early as 1980. This investment contributed to the expansion of BSL-3 laboratory infrastructure, including facilities reported to focus on the study of transmission mechanisms of viruses causing epidemic hemorrhagic fevers [20,21]. Similarly, several USA states have recognized the need for investment in biodefense facilities. This concern gained significant momentum following the terrorist attacks in 2001, which prompted the USA government to allocate substantial funding for biodefense infrastructure [22].

Additionally, epidemics such as the SARS outbreak in China between 2002 and 2003 stimulated governmental efforts to improve biosecurity and expand biosafety infrastructure. The epidemic, which resulted in 8098 cases and 774 fatalities, highlighted the urgent need to strengthen the country’s public health system [19]. Following the outbreak and several globally reported cases of laboratory-acquired infections, biosafety became a top priority for both central and local Chinese governments [23]. A series of biosafety-driven policies were implemented to support legislative reforms and promote the construction of additional high-level biosafety laboratories [24]. This shift towards policy and standardization marks a critical evolution from ad hoc responses to the establishment of a systemic framework for quality and risk management, aiming to ensure consistency and compliance across all facilities [25]. This period marked a turning point in the sustainable operation and strategic management of biosafety infrastructure in China [19].

These efforts may have contributed to increased investment and interest in R&D in this area. As shown in Figure 2B, the estimated number of patents expiring between 2024 and 2028 reflects the applications filed during that period, given that they generally expire after approximately 20 years [26].

In the later years, greater attention was devoted to the regulation of biosafety and biosecurity laws. In 2004, the World Health Organization (WHO) published a manual outlining recommendation for the structure and operation of BSL-3 facilities [27]. Furthermore, each country has developed its own biosafety legislation [28]. This period of legal consolidation may help explain why, in 2009, the lowest proportion of patents related to BSL-3 technologies was recorded (only 1.72% of the total). This low point in patent activity can be interpreted as a consolidation phase within the quality management system. Organizations were likely prioritizing the internalization, training, and implementation of new international and national standards, which temporarily slowed down disruptive innovation as the focus shifted to compliance and integration [29]. However, this number increased between 2011 and 2014, with both years registering 3.45% of the total patents. This growth may have been related to the accreditation of high-level biosafety laboratories in China: by 31 December 2013, 53 BSLs, including 42 BSL-3s, had been fully accredited [19]. Other contributing factors likely include the 2014 Ebola outbreak [30] and the growing international concern over bioterrorism threats [31].

In 2016, a notable increase was observed, with 6.90% of the patents registered in that year. This was followed by relatively stable numbers in 2017 (5.17%) and 2018 (6.90%) (Figure 2A). Additionally, a significant increase in patent registrations related to BSL-3 technologies was observed in 2019, accounting for 15.52% of the total (more than double compared to previous years). This surge can be attributed to the emergence of the COVID-19 pandemic, which prompted global mobilization in public health and research [32]. In this context, BSL-3 laboratories are specifically designed to handle serious or potentially lethal pathogens for which vaccines or treatments may be available (such as coronaviruses, the causative agents of COVID-19) [33]. The increase in R&D investment during this period is strongly associated with the urgent demand for vaccine development and pathogen containment strategies [34].

Moreover, scenarios involving virus cultures, potential exposure to infectious aerosols, the emergence of highly transmissible variants, and zoonotic risks from laboratory animals necessitate the implementation of advanced BSL-3 measures [6]. In this sense, the growing global interest in BSL-3 technologies has led to the construction of laboratories not only in China and the United States (leaders in the development of these technologies), but also in countries such as Brazil, India, and Japan [35,36]. The sustained focus on BSL-3 infrastructure and technology remained evident through 2020, driven by the pressing need to safely manipulate SARS-CoV-2 and by the strong interest of both the pharmaceutical industry and governments in R&D investment [37]. Given that COVID-19 pathogens must be handled in BSL-3 settings, this demand has directly contributed to the spike in related patent activity.

Briefly, in 2021, a reduction in patent filings was observed (10.34%), which may be partially explained by the release of effective COVID-19 vaccines in late 2020 and early 2021, diminishing the urgency for rapid technological development. However, the continued construction of BSL-3 laboratories in emerging economies and the interest of these countries in developing domestic vaccine capabilities likely contributed to the relatively high number of patent filings in 2022 (13.79%) when compared to pre-pandemic years. Moreover, some institutions implemented adaptations to convert existing BSL-2 laboratories into BSL-3-compatible facilities, which may have reduced the need for new infrastructure-related innovations, particularly in construction and equipment design [38].

Since 2023, a gradual decline in patent activity related to BSL-3 technologies has been observed—6.9% in 2023 and 8.62% in 2024 (Figure 2A). Nevertheless, this trend may represent an opportunity for investors and innovators. As many developing countries have shown increasing interest in the development of BSL-3 laboratory infrastructure, there is a growing market for accessible and cost-effective technologies tailored to non-consolidated settings. Investment and innovation in this area could play a key role in strengthening global biosecurity. Finally, a considerable proportion of existing patents in this domain are expected to expire by 2029 (20.45%) (Figure 2B), potentially opening strategic windows for governments and private sectors to expand R&D initiatives and implement next-generation biosafety solutions.

### 3.2. China Dominates the Global Patent Landscape for BSL-3 Technologies, While Emerging Economies Present Opportunities for Future Innovation

**Figure 3:**
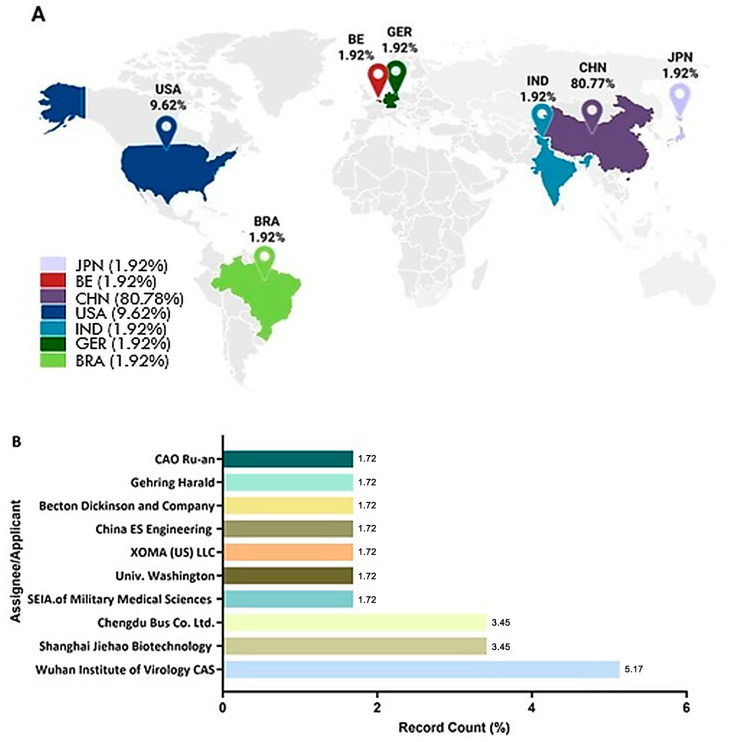
**Global monitoring of patent applications.** Distribution of patents by filing origin (**A**) and by institutional assignees/applicants (**B**). Univ. Washington: University of Washington; SEIA of Military Medical Sciences: Sanitary Equipment Institute Academy of Military Medical Sciences PLA.

Another indicator for analysis in this outlook study is the geographical distribution of patent filing activity related to BSL-3 technologies. Patent filings were used as indicators of technological innovation and intellectual property activity rather than as direct measures of the implementation, deployment, or operational use of BSL-3 laboratories. The analysis of patent documents in this field revealed a pronounced concentration of intellectual property activity in China, which accounted for approximately 80.77% of the retrieved patents, positioning the country as the global leader in technological developments related to BSL-3 laboratories. The United States followed with 9.26% of filings, while Brazil, Japan, and India each contributed around 1.92% of the total patents (Figure 3A). Only countries represented in the final patent dataset are shown in Figure 3A. Countries not displayed were not represented in the retrieved patent records after applying the search strategy and eligibility criteria.

The predominant leadership of China in the global patent landscape related to biosafety and biosecurity may be associated with the country’s long-standing governmental investment and its strategic regulatory framework in these areas. In fact, since the 1980s, China has shown an interest in the development of biosafety legislation aimed at regulating and supporting advancements in biotechnology [39].

The emergence of SARS-CoV-2 further accelerated China’s commitment to biosafety [40]. In response to the pandemic, the country significantly increased its investments not only in laboratory containment, but also in aerosol control systems and pathogen inactivation technologies essential for the operation of BSL-3 laboratories. This includes the development of secure environments required for vaccine production and virus manipulation [38]. It is important to note that China’s investments in high-containment laboratory infrastructure predate the COVID-19 pandemic. For instance, the country inaugurated its first BSL-4 laboratory in Wuhan in 2015, which was accredited by the China National Accreditation Service for Conformity Assessment (CNAS). This demonstrates that China has long been interested in the technological area of high-level biological safety laboratories [41].

China has also adapted its national biosafety laws to address the country’s priorities as well as international expectations. The enactment of the Biosafety Law in 2021 marked a milestone in the institutionalization of biosafety governance in China. Although this legislative process was initiated before the pandemic, it was catalyzed by the global health crisis and aimed to unify fragmented policies in areas such as laboratory safety, biotechnology regulation, and genetically modified organisms (GMOs), thereby contributing to a more coherent and integrated governance system [42,43]. Taken together, these factors may have contributed to China’s rise to leadership in biosafety-related patent activity. This leadership can be attributed not only to early and sustained investment in biotechnological infrastructure, but also to the strategic incorporation of biosafety into broader frameworks of national security and international influence, as reflected in national policies concerning biosafety and biosecurity [22].

On the other hand, the United States (USA) accounted for only 9.26% of the patent filings identified in this study. This outcome is atypical for the USA, which traditionally stands at the forefront of global technological advancement. While the COVID-19 pandemic catalyzed BSL-3 technological advancements in China, the opposite trend was observed in the United States. In addition, this scenario of low production may be linked to disruptions caused by the COVID-19 pandemic, including laboratory closures, delays in R&D activities, a reduction in the launch of new research projects, and the reallocation of resources to urgent pandemic-related priorities. This trend may have temporarily influenced the pace of patent filings specifically targeting high-containment laboratories, including technologies associated with BSL-3 environments.

Similarly, India, Brazil, and Japan each account for only 1.92% of the patent filings related to this technological domain. This limited representation may be associated with the absence of consistent governmental investment in biosafety, or with public policies that are not conducive to the establishment of such infrastructure [44,45,46,47,48]. In practice, the bureaucratic hurdles involved in developing this type of technology can be perceived by investors as deterrents, slowing down the implementation process [49]. However, the low number of patents in these countries may also suggest the existence of future opportunities: as research efforts in these regions expand, a growing demand could emerge, potentially creating a favorable environment for future investment.

Indeed, most assignees and applicants are concentrated in China (Figure 3B). Among the ten most prevalent applicants, nine are Chinese, accounting collectively for 22.39%, with the Wuhan Institute of Virology (CAS) alone representing 5.17%. In contrast, only one of the most frequent assignees is based in the USA, the University of Washington, contributing 1.72% of all filings. Overall, applicants from China represent approximately 80% of the total submissions (Supplementary Table S1). This concentration suggests that China already possesses a strong base of investors, while other countries may represent promising opportunities for assignees and applicants interested in investing to fill the existing gaps in a technological sector that is not yet fully consolidated.

It is important to note that the geographic distribution of patent filings may also reflect differences in national patent systems, public investment in research and development, and biosafety regulatory frameworks. These country-specific factors can influence both the capacity to generate technological innovations and the propensity to seek patent protection. Therefore, the observed distribution of BSL-3-related patents should be interpreted as an indicator of innovation activity within different national contexts rather than as a direct comparison of technological capability or biosafety infrastructure across countries.

### 3.3. Air Filtration and Biotechnology Dominate BSL-3 Patent Technologies, While Automation and Wastewater Treatment Remain Underexplored

Understanding the technological areas of interest and market demands is essential to align innovations with both commercial and scientific goals. In this context, technologies can be grouped and classified according to the International Patent Classification (IPC) codes, which serve as valuable tools for identifying technological trends and gaining insight into the functional scope and intended applications of BSL-3-related innovations. Table 1 summarizes the main IPC codes found in the analyzed patent documents, along with their respective descriptions.

The most prevalent technological trends identified among the patent documents were related to IPC class B01, which accounted for 20.10% of the analyzed codes, followed by C12 (12.98%) and A61 (12.21%). In contrast, the least frequent IPC classes were B09 and H04, each representing only 1.53% of the technological area addressed in the patents.

B01 refers to general physical or chemical methods and apparatuses, such as filtration, mixing, and separation; its prominence may be attributed to the operational requirements of BSL-3 laboratories. These facilities handle infectious microorganisms that pose a serious or potentially lethal risk via aerosol transmission, and therefore, they require advanced filtration systems, including powered air-purifying respirators (PAPRs) fitted with HEPA filters. Compliance with biosafety regulations necessitates such systems, which may explain the significant number of patent filings in this technological class [50]. From a quality management perspective, the high volume of B01 patents reflects a critical focus on standardizing and optimizing core engineering controls. This indicates a mature understanding that robust, reliable processes are the foundation of risk mitigation and quality operations in a high-containment environment [51].

Similarly, C12, which encompasses biochemistry, microbiology, enzymology, and genetic engineering, features prominently among the patent classifications. This is likely due to the central role these disciplines play in the work conducted within BSL-3 environments. These laboratories are specifically designed for the manipulation of microorganisms, including genetically modified ones, requiring innovative devices, protocols, and processes for containment, decontamination, and personal protection [52].

The A61 class, related to medical or veterinary sciences and hygiene, includes inventions such as sterilization methods, dressing materials, and veterinary instruments. These are particularly relevant in high-containment biological laboratories (HCBLs), which support research involving Risk Group 3 and 4 pathogens across BSL-3, BSL-4, ABSL-3, BSL-3-Ag (agricultural livestock), and ABSL-4 laboratories [6].

**Figure 4:**
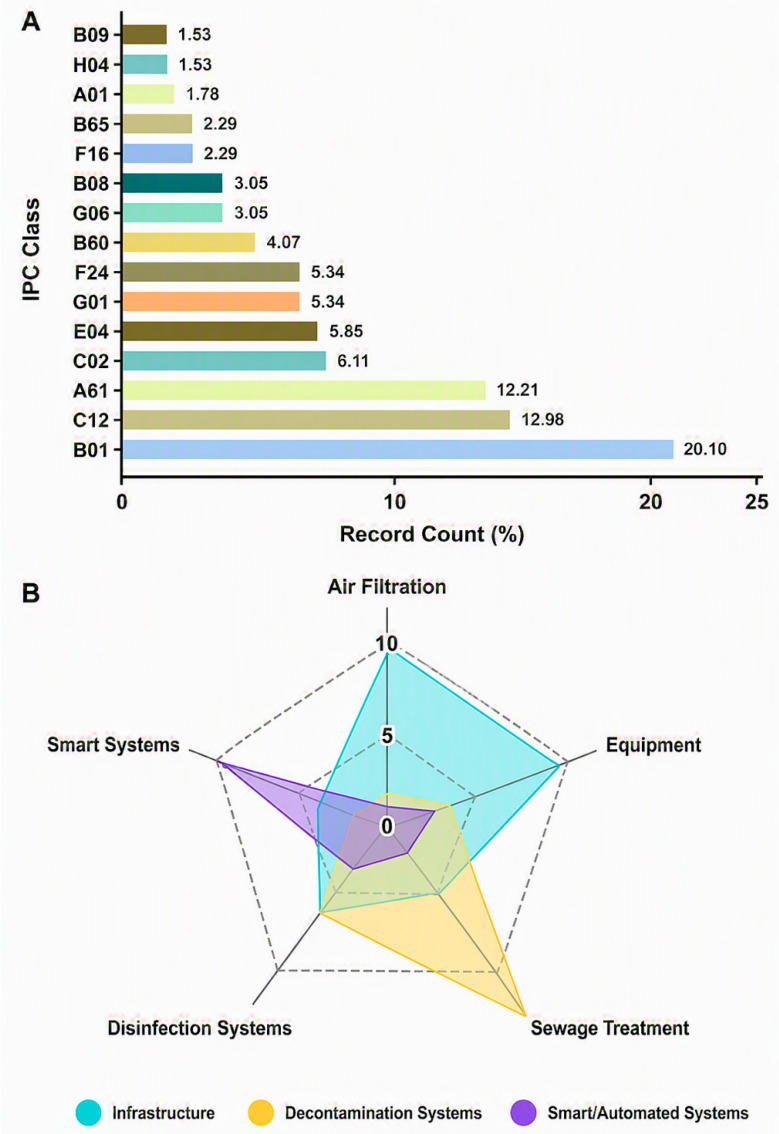
**Technological distribution and innovation focus of IPC classes in BSL-3-related patents.** Frequency of the most common IPC classes (**A**) and comparison of the technological focus areas across the most prevalent IPC classes (**B**).

Moreover, the design, construction, and maintenance of BSL-3 require substantial investment and are essential for sustaining research, development, and response capabilities. These laboratories are vital for handling high-consequence pathogens and supporting all stages of research—from basic to translation. Although IPC classes such as B09 (waste treatment and disposal) and H04 (electronic communication technologies) are less represented in patent data, their relevance should not be underestimated. BSL-3 operations require secure systems for waste management and data transmission, indicating that these are underexplored, yet critical, areas for future technological development and investment opportunities (Figure 4A).

Furthermore, an analysis of the technological fields represented in the patent dataset revealed that air filtration and equipment-related technologies have received the greatest attention from inventors, as reflected by the highest values in IPC subgroups (Figure 4B). These domains are fundamental for the proper functioning of BSL-3 laboratories, particularly due to the need for high-efficiency filtration systems and specialized apparatuses to ensure containment and operational safety.

In contrast, wastewater treatment, disinfection systems, and smart/automated systems appear less frequently among the analyzed patents, suggesting a comparatively lower level of technological investment in these areas. This underrepresentation may indicate unmet needs or technological gaps in the development of advanced decontamination methods, automation tools, and digital control systems for high-biosafety environments. Consequently, these areas may represent promising opportunities for innovation and investment, especially as the demand for modern, responsive, and resilient biosafety infrastructures continues to grow.

### 3.4. Air Filtration, Laboratory Equipment, and Microbiological Systems Dominate Patented BSL-3 Infrastructure Technologies

The patent document analysis revealed that 20% of the examined patents were classified under IPC code B01, indicating a strong focus on technologies related to general physical or chemical methods, including filtration, separation, and purification systems. A single patent may fall under multiple IPC codes, and in this study, the B01 classification appeared 79 times, underscoring its prominence in biosafety-level infrastructure.

This technological trend is exemplified by CN101711935A, which describes a high-efficiency air filtration unit designed for leak detection and local disinfection. The system integrates automatic scanning mechanisms, disinfection devices, and HEPA filter monitoring, ensuring compliance with biosafety standards while enabling both air intake and exhaust treatment in advanced biosafety laboratories. Similarly, US9446159B2 discloses a flow cytometer system equipped with a biosafety hood (BSH) and an aerosol management system, emphasizing redundant filtration mechanisms to prevent the release of hazardous aerosols, thereby enhancing laboratory safety during flow-cytometric procedures. Moreover, DE202015008363U1 presents a depressurization flash tank with biological containment features, incorporating pressure regulation, rupture disks, sight glasses, disinfection ports (CIP/SIP), and sterile filtration units. Its design specifically prevents the uncontrolled release of biological agents, ensuring both personnel safety and environmental protection.

The second-most frequent technological area identified during the prospection corresponds to IPC class C12, which encompasses technologies in biochemistry, microbiology, and enzymology. This classification occurred 51 times among the analyzed patent documents, reflecting the relevance of biotechnological and microbiological processes in BSL-3 infrastructure. Representative examples include CN206768091U, which describes a biosafety laboratory operating platform equipped with integrated drainage, sterilization, and wastewater treatment systems. The platform’s perforated surface enables controlled liquid drainage, directing effluents to a sterilizing tank connected to a sewage treatment unit, thereby enhancing biosafety and hygiene during experimental procedures. CN210237599U details a pre-vertical fermentation unit designed for sewage treatment systems in biosafety laboratories. The apparatus incorporates a heating rod, stirring shaft, spiral crushing blades, and dual-valve control, enabling the efficient processing, fermentation, and sterilization of laboratory effluents. Its structural versatility supports both operational efficiency and environmental safety.

For instance, the frequency of A61 (medical or hygiene-related technologies) is 48, reflecting the relevance of health-focused innovations in BSL-3 environments. C02 (water and wastewater treatment) appears 24 times, underscoring the importance of effective decontamination and effluent management systems. E04 (building structures and civil engineering) is present in 23 documents, highlighting interest in specialized architectural solutions for containment and safety. G01 (measuring and testing instruments) and F24 (heating and ventilation) both occur 21 times, indicating demand for precision monitoring tools and optimized air-handling systems.

Additionally, B60 (transport systems) appears in 16 documents, followed by G06 (data processing and artificial intelligence) and B08 (cleaning technologies), each with 12 occurrences. Technologies related to F16 (mechanical fittings) and B65 (handling and storage) were found nine times each. Less frequent, but still relevant, are A01 (agriculture/biological materials), with seven patents, and H04 (communication technologies) and B09 (waste disposal and environmental protection), each with six occurrences. A detailed breakdown of these classifications is presented in Table 2 and Supplementary Table S2.

Furthermore, a more detailed analysis of the current IPC groups demonstrated that the B01 codes are distributed across subcategories such as B01D/46 (filtration by physical methods) and B01L/00 (laboratory equipment), highlighting the predominance of technologies related to air filtration and laboratory devices intended for handling biological materials and environmental factors. Moreover, the high frequency of B01L/00 in the IPC code registrations underscores the strong interest of assignees in the adaptation of laboratory equipment and surfaces to meet biosafety requirements. This trend may be associated with the operational demands of BSL-3 laboratories, which prioritize contamination control and the safe handling of pathogens. BSL-3 facilities are considered an essential component of the public health response to emerging infectious diseases, particularly in the context of the COVID-19 pandemic, which underscored the importance of biosafety infrastructures for metagenomic surveillance, disease prevention, and control [48]. Among the C12 IPC classes, the subcategories of particular interest are C12M 1/00 and C12M 3/06. These classifications are associated with technologies aimed at developing devices applicable to microbiological processes within containment systems.

In addition, the E04 category (related to infrastructure and construction) shows prominent activity in the subcategories E04H 12/00 and E04H 8/00 (Figure 4). The increased attention to infrastructure-related technologies may also be linked to the global rise in emerging infectious diseases in recent years [36]. Additionally, the pathogens involved in such events are classified as high-consequence pathogens, due to their serious risks to both human and animal health. Moreover, the potential misuse of these pathogens for antagonistic purposes, such as bioterrorism, further underscores the critical need for national preparedness [52]. Although documented cases of bioterrorist attacks remain limited, various extremist groups have publicly expressed such intentions, reinforcing the urgency for early detection systems and robust containment strategies in biosafety infrastructure [53].

Other relevant categories include F24 (ventilation systems), particularly subcategories F24F 6/14, which refers to ventilation systems with temperature or humidity control, and F24F 11/89, which addresses automated ventilation control systems. These classifications reinforce the growing trend of research and development (R&D) focused on smart, automated technologies designed to regulate environmental conditions in BSL-3 laboratories; such systems are associated with rapid and adaptive responses to environmental changes, thereby enhancing biological safety.

**Figure 5:**
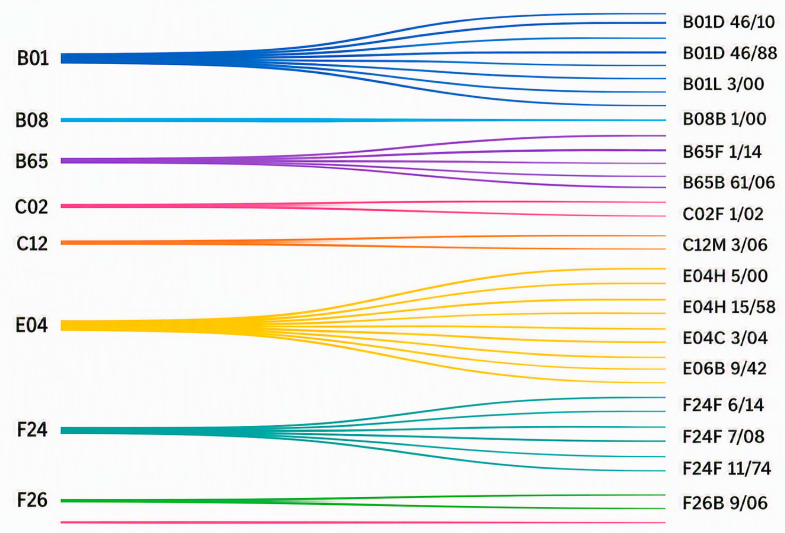
**Air filtration, laboratory equipment, ventilation, and microbiological systems are the predominant IPC subcategories in BSL-3-related patents.** Highlighting ventilation (F24F), filtration (B01D), lab equipment (B01L), microbiological systems (C12M), and infrastructure (E04H), with fewer entries in cleaning (B08), storage (B65), and drying (F26).

On the other hand, technologies such as B08 (cleaning), B65 (handling and storage), and F26 (drying) appear less frequently in the analyzed patents (Figure 4). Although these technologies are essential, especially in the context of sterilization, transportation, and contaminated material processing, their low patent representation may suggest that these functions are more commonly embedded within broader technological classes related to BSL infrastructure. Nevertheless, considering their critical importance in the operational safety of BSL-3 laboratories, the limited number of patents in these areas may reflect an underexplored technological niche. This scenario could represent a promising opportunity for new patent applications and innovation, particularly given that the WHO Laboratory Biosafety Manual (LBM) highlights cleaning and sterilization practices as non-optional components of biosafety protocols in BSL-3 laboratories [54] (Figure 5).

Overall, the patent landscape indicates that current BSL-3 technological innovations are primarily focused on improving biological containment, air filtration, laboratory equipment, and operational safety, thereby enhancing the reliability and performance of high-containment laboratories. However, the relatively limited number of patents related to cleaning technologies, waste management, automated control systems, and material handling suggests that these areas remain underexplored. These technological gaps may represent promising opportunities for future research, development, and innovation aimed at improving the efficiency, sustainability, and resilience of BSL-3 laboratory infrastructure.

## 4. Conclusions

This study provided a comprehensive patent landscape analysis of technologies related to Biosafety Level 3 (BSL-3) laboratories, identifying the main technological trends, leading countries, patent applicants, and innovation areas associated with high-containment laboratory infrastructure. The results revealed a strong concentration of patent activity in China, followed by the United States, with technological developments primarily focused on air filtration systems, laboratory equipment, microbiological processes, and infrastructure. Conversely, technologies related to cleaning systems, waste management, automation, and material handling were comparatively underrepresented, suggesting opportunities for future innovation.

Due to strict international standards, BSL-3 technologies represent a strategic opportunity for government and private investments. Although China and the United States remain the most consolidated players in the biosafety technology market, these countries continue to invest in the sector, driven by growing concerns over pandemics and bioterrorism, especially after COVID-19. The expansion of interest in BSL-3 laboratory infrastructure in developing countries highlights emerging markets and increasing global demand. As patent expirations approach, especially around 2029, new opportunities are likely to arise for innovation, technology transfer, and international collaboration in the field of high-containment laboratories. This patent cliff is not a merely market event, but a trigger for a new cycle of continuous improvement. It opens the door for competition, which can drive down costs, increase accessibility, and create space for new technologies that offer efficiency, integration, and user safety, fostering a new wave of strategic innovation for high-containment laboratories. These findings may support strategic decision making by governments, research institutions, and private companies seeking to strengthen biosafety infrastructure and foster innovation in high-containment laboratory technologies.

## Figures and Tables

**Table 1:** Main IPC codes identified in BSL-3-related patent documents and their technological descriptions.

IPC Code	Technological Description
B01	General physical or chemical methods and apparatus (e.g., filtration, mixing, separation)
C12	Biochemistry; microbiology; enzymology; genetic engineering
A61	Medical or veterinary science; hygiene
C02	Treatment of water, wastewater, sewage, or sludge
E04	Building construction
G01	Measuring; testing
F24	Heating; ventilation
B60	Vehicles in general
G06	Computing; calculation; counting; data processing
B08	Cleaning; cleaning-in-place (CIP) systems
F16	Engineering elements or units (e.g., pipes, joints, valves)
B65	Conveying; packing; storing; handling thin or filamentary material
A01	Agriculture; forestry; animal husbandry
H04	Electric communication techniques (e.g., transmission of digital information)
B09	Waste disposal; waste treatment or reuse

**Table 2:** Description of patent documents related to BSL-3 technologies.

Publication No./Date	Title	Technology	Country	IPC Class	Assignee
CN101711935A/2009-10-19	High Efficiency Air Filter Unit	Ventilation: HEPA filtration unit	CN	B01	Lanzhou Veterinary Research Institute, Chinese Academy of Agricultural Sciences, Lanzhou, Gansu, China./Institute of Medical Equipment, Academy of Military Medical Sciences, Tianjin, China
US8137615B2/2011-01-14	Automated Workstation for Disinfecting Objects and Methods of Use Thereof	Equipment: Disinfection workstation	US	A61, A01	University of Washington, Seattle, WA, USA
US10294658B2/2011-07-13	Flexible Manufacturing System	Infrastructure: Modular manufacturing system	US	C02, B23, E04, G05	Xoma Corporation, Berkeley, CA, USA
CN103848462B/2014-01-14	BSL-3 Laboratory Wastewater Centralized Processing System|BSL-3	Waste Management: Centralized wastewater system	CN	C02, G01	China Electronics System Engineering No. 2 Construction Co., Ltd., Wuxi, Jiangsu, China
US9446159B2/2014-10-02	Flow Cytometer Biosafety Hood and Systems Including the Same	Analytical Equipment: Flow cytometer with biosafety hood	US	A61, B01, G01	Becton Dickinson and Company (BD), Franklin Lakes, NJ, USA
DE202015008363U1/2015-12-03	Depressurization Flash Tank with Water Trap or Intermediate Diaphragm for Safety Devices with Biosafety Level (BSL) 1-2-3 And 4 Regions	Containment Equipment: Pressure relief container	DE	B65, B01	Gehring Technologies GmbH, Ostfildern, Germany
CN105865870A/2016-04-12	Specimen Pretreatment Equipment	Lab Equipment: Specimen pretreatment machine	CN	G01, B65	Cao R
CN106245944B/2016-06-15	Analyzing And Detecting the Base Laboratory Building Module	Construction Module: Base lab unit with air purification	CN	E04	Gao H
IN404564B/2016-06-20	Portable Decontamination Unit	Decontamination: Biosafety cabinet sterilization system	IN		American Sterilizer Company |Steris Corporation, OH, USA
CN205974094U/2016-08-29	Laboratory Sewage Treatment Device	Waste Management: Sewage treatment box	CN	C02	Beijing Anyutong Environmental Engineering & Technology Co., Ltd., Beijing, China
CN206768091U/2017-05-31	A Biological Safety Laboratory Experiment Operating Platform	Workstation: Biosafety experimental platform	CN	C12	Institute of Medical Biology Chinese Academy of Medical Sciences, Kunming, Yunnan, China
BE1025019B1/2017-07-28	Assembly Comprising a Framework and at Least One First Element to Be Connected	Infrastructure: Cleanroom ceiling assembly	BE	E04	Becarv Sa, Buenos Aires, Argentina
CN207877519U/2017-12-29	Integrated Laboratory Sewage Treatment Device	Waste Management: Integrative sewage system	CN	C02	Guangxi Bossco Environmental ProtectionTechnology Co., Ltd., Nanning, Guangxi, China
CN108328817A/2018-02-01	A Concentrated-Processing Laboratory Sewage Treatment Device	Waste Management: Centralized processing system	CN	C02	Jiangsu Kulinan Laboratory Equipment Co., Ltd., Taizhou, Jiangsu, China
CN208462175U/2018-06-27	A Biological Safe Laboratory Full Automatic Comprehensive Control Cabinet	Control Systems: Automated control cabinet	CN	H05	Beijing Cleanair Biological Laboratory Engineering Co., Ltd., Beijing, China
CN210217221U/2018-09-27	Integrated Laboratory System	Infrastructure: Integrated laboratory system	CN	E04, B01	Suzhou Purification EngineeringInstallation Co., Suzhou, Jiangsu, China
CN209261264U/2018-11-28	A Shelter Combined BSL-3 Laboratory	Animal Facilities: BSL lab with IVC cage and exhaust filter	CN	E04, A01, F24	Zhenjiang Kangfei Automobile Manufacturing Co., Ltd., Zhenjiang, Jiangsu, China
CN209673388U/2019-02-26	A Quantitative Sealing Excrement Special Collection Tube	Sampling Tools: Sealed excrement collection pipe	CN	G01	Changsha Xieda Biological Technology Co, Changsha, Hunan, China
CN210237599U/2019-06-25	Pre-Vertical Fermentation Structure Applied to the Sewage Treatment System of Biosafety Laboratory	Waste Management: Fermentation structure for sewage treatment	CN	C12	Nanjing Chuanye Environmental Protection Technology Co., Ltd., Nanjing, Jiangsu, China
CN110216719B/2019-07-05	A Biological Experiment Protective Device for Biological Safety Laboratory	Protection: Biosafety lab protective gear system	CN	B25, B01, B08	China Academy of Building Research Co, Ltd.| North China Electric Power University, Beijing, China
CN210736423U/2019-08-28	Integrated Laboratory Sewage Comprehensive Processing Device	Waste Management: Comprehensive sewage device	CN	C02	Shandong Bsd Environmental Protection Technology Co., Ltd., Jinan, Shandong, China
CN210656591U/2019-08-30	Combined Laboratory Sewage Comprehensive Processing Device	Waste Management: Combined sewage treatment unit	CN	C02	Shandong Aokunlai Intelligent Technology Co., Ltd., Jinan, Shandong, China
CN211382920U/2019-12-04	A Biological Safety Laboratory Fog Shower Room	Decontamination: Fog spraying chamber	CN	A61, F26	Shanghai Jiehao Biotechnology Co, Ltd., Shanghai, China
CN211755061U/2019-12-16	A Biological Safety Laboratory Flume	Infrastructure: Ferry groove with fumigation interface	CN	B01	Shanghai Jiehao Biotechnology Co, Ltd., Shanghai, China
CN211612743U/2019-12-26	A Biological Safety Cabinet	Workstation: Biosafety cabinet with waste barrel	CN	B01	Beijing Xinji Yongkang Biological Technology Co., Ltd., Beijing, China
CN211370144U/2019-12-31	Airtight Door for Biological Safety Laboratory	Infrastructure: Airtight lab door with sealing ring	CN	E06	Academy of Military Sciences, Academy of System Engineering Medicine, Beijing, China|Tianjin Changte Purification Engineering Co, Ltd., Tianjin, China
CN211508486U/2020-03-17	A Special Device Belt for Biological Safety Laboratory	Cabling Infrastructure: Equipment belt with disinfection holes	CN	H02	Ippr Lab System Technology Co., Beijing, China
CN111502350A/2020-03-27	Air Inflation Membrane Structure Virus Detection Laboratory, And Covering Structure Virus Detection Laboratory	Infrastructure: Inflatable virus detection lab	CN	E04, F24	Bgi Genomics Co, Ltd., Shenzhen, Guangdong, China |Bgi Shenzhen Co, Ltd., Shenzhen, Guangdong, China| University Tongji, Shanghai, China
CN116651536A/2020-04-16	Biosafety Laboratory	Infrastructure: Inflatable structure for biosafety lab	CN	B01	Bgi Shenzhen Co, Ltd., Shenzhen, Guangdong, China |Shanghai Etopia Building Technology Co., Ltd., Shanghai, China
JP03242451U/2020-04-16	Inflatable Film Structure Virus Test Laboratory; Biosafety Test Laboratory; Topsoil Type Structure Virus Test Laboratory	Ventilation: Inflatable lab with heat exchange system	JP	E04	Bgi Genomics Co., Ltd., Shenzhen, Guangdong, China
BR202020014109U2/2020-07-09	Resistant Thermo-Resistant Tripsnizer Assembly with Threadable Hermetic Seal	Analytical Equipment: Trypsinizer for vaccine production	BR	B01	Adilson S
CN111913455B/2020-08-13	An Intelligent Comprehensive Control System of Biological Safety Laboratory	Control Systems: Intelligent integrated monitoring system	CN	G05	Suzhou Huatuo Biotechnology Co., Ltd., Suzhou, Jiangsu, China
CN111957356A/2020-08-13	Movable Biological Safety Laboratory	Infrastructure: Movable BSL lab with support and experiment cabins	CN	B01	Yinlong New Energy Co., Ltd., Zhuhai, Guangdong, China|Zhuhai Guangtong Automobile Co., Ltd., Zhuhai, Guangdong, China
US12005432B2/2020-10-08	Configurable Workstations	Workstation: Modular biological protocol station	US	G01, B01, C07, C12, G05, G06, G16, H04	Coopersurgical Inc., Trumbull, CT, USA
CN112140976A/2020-10-23	Self-Propelled Biological Safety Laboratory	Infrastructure: Self-propelled BSL lab with HVAC	CN	B60	Chengdu Bus Co., Ltd., Chengdu, Sichuan, China
CN214083781U/2020-10-23	Ventilation Pipeline System of Self-Propelled Biological Safety	Ventilation: Mobile BSL ventilation pipeline system	CN	B60	Chengdu Bus Co., Ltd., Chengdu, Sichuan, China
CN214620347U/2021-03-03	Biological Safety Laboratory Sterilizing Device Convenient for Installation	Decontamination: Lab sterilizing box with UV	CN	F26, A61	Nanjing Bosen Technology Co., Ltd., Nanjing, Jiangsu, China
CN214581692U/2021-03-15	An Air Sterilizing Device for Biological Safety	Air Sterilization: Biological safety air sterilizer	CN	F24	Shenzhen Xige Industry Co., Ltd., Shenzhen, Guangdong, China
CN113006542A/2021-04-29	Biological Safety Secondary Laboratory Based on Negative Pressure Tent	Containment: Negative pressure tent lab	CN	E04, E06	Chongqing Oriental data Technology Co., Ltd. Chongqing, China
CN215314614U/2021-06-30	A Biological Safety Cabinet	Workstation: Biosafety cabinet with sterilizing box	CN	B08, A61, B01	Hainan Viewkr Bio-Tech Co., Ltd., Haikou, Hainan, China
CN216223402U/2021-11-12	Biological Safety Working Table with Hepa Filtering System and Ultraviolet Irradiation Device	Workstation: Biosafety working table with HEPA and UV	CN	B01, A61	Dream Lab Technology (Shanghai) Co., Ltd., Shanghai, China
CN113914441A/2021-11-25	Floor Drain for High Grade Biological Safety	Drainage: Lab floor drain for high-level biosafety	CN	E03, B02	Wuhan Virology Institute, Chinese Academy of Sciences, Wuhan, Hubei, China
CN114408414A/2022-01-13	A Biological Safety Protecting Device for Biological Safety	Protection: Film-based safety device for labs	CN	B65	The Eighth Medical Center, Chinese PLA General Hospital (Beijing, China)
CN116115803A/2022-09-07	A Multifunctional Safe Chemical Shower Disinfecting System and Method	Decontamination: Multifunctional sterilizing shower system	CN	A61	Wuhan Virology Institute, Chinese Academy of Sciences (CAS) (Wuhan, Hubei, China)
CN218530958U/2022-10-14	Biological Safety Cabinet with Intelligent Exhaust Device	Workstation: Sealed biosafety cabinet with movable parts	CN	B01	Shanghai Radobio Science Co., Ltd., Shanghai, China
CN115466674A/2022-10-27	Biological Sampling and Culturing Device for Biological Safety Laboratory	Culture Equipment: Biological sampling and cultivation system	CN	C12	Ippr Lab System Technology (Beijing) Co., Ltd., Beijing, China
CN218912384U/2022-12-09	Full-Automatic Pcr Square Cabin Detection Laboratory	Infrastructure: Full-automatic PCR square cabin	CN	E04, C12	Chongqing Bluehorizon Energy Saving Technology, Co., Ltd. (Chongqing, China)
CN116105272A/2022-12-20	Negative Pressure Control System of High-Grade Biological Safety Laboratory and Micro-Negative Pressure Sterilizing Method	Control Systems: Negative pressure control system	CN	F24	Harbin Veterinary Research Institute, Chinese Academy of Agricultural Sciences (CAAS) (Harbin, Heilongjiang, China)
CN219494298U/2022-12-26	An Exhaust System for Biosafety Laboratory	Ventilation: Exhaust system with dual valves	CN	F24	Shaanxi Meili-Oh Animal Health Co., Ltd., Xi’an, Shaanxi, China
CN116065734A/2022-12-28	Method For Setting Enclosure Structure of High-Grade Biological Safety Laboratory	Construction: Enclosure structure for BSL	CN	E04	Shanghai Hushi Laboratory Equipment Co., Ltd., Shanghai, China
CN116273215A/2023-04-27	A Hepa Filter Box Body with Sealed Original Position and Using Method Thereof	Ventilation: HEPA filter box with sealed sterilization	CN	B01	Wuhan Virology Institute, Chinese Academy of Sciences (CAS), Wuhan, Hubei, China
CN221016102U/2023-08-30	Conveyor Belt Type Bio-Safety Cabinet	Workstation: Conveyor belt biosafety cabinet	CN	B01, A61	Zhongke Meiling Cryogenics Co., Ltd., Hefei, Anhui, China
CN221384165U/2023-10-31	Biosafety Negative Pressure Dissecting Table	Workstation: Negative pressure dissecting table	CN	A61	Wuxi Lamoton Technology Co., Ltd., Wuxi, Jiangsu, China
CN221122506U/2023-11-13	Air Conditioning System and Tent Biological Safety Laboratory	Ventilation: Tent lab air conditioning with filtration	CN	F24	Tianjin Hanaco Medical Co., Ltd., Tianjin, China
CN118066628A/2024-01-23	Ventilated Air Filtration Sterilization Method and Related Apparatus	Ventilation: Cleanroom filtration and sterilization system	CN	F24	Shenzhen Wanwei Air Conditioning Purific Co., Ltd., Shenzhen, Guangdong, China
CN118253560A/2024-04-16	Air Exhausting and Purifying Device for Biosafety Laboratory	Ventilation: Exhaust air purification unit	CN	B08	Guangzhou Lanjing Environmental Technology Co., Ltd., Guangzhou, Guangdong, China
CN222287342U/2024-05-24	Biological Safety Cabinet with Stable Structure	Workstation: Biosafety cabinet with screen plate	CN	B01	Hainan Chuanyi Industry Co., Ltd., Haikou, Hainan, China
CN119330234A/2024-11-01	Lifting Device for Biosafety Laboratory	Lifting Equipment: Lab hoisting device	CN	B66	Military Veterinary Research Institute Pla Academy of Military Sciences, Changchun, Jilin, China

## Data Availability

The original contributions presented in this study are included in the article and Supplementary Materials. Further inquiries can be directed to the corresponding author.

## Supplementary Materials

The following supporting information can be downloaded at: https://www.mdpi.com/article/10.3390/biotech15030052/s1, Table S1: Top patent applicants in BSL-3 technologies, showing leading institutions and companies, with their respective percentage share of total filings. Table S2: Description of patent documents related to BSL-3 technologies.

## Abbreviations

The following abbreviations are used in this manuscript:

BSL-3	Biosafety Level 3
DWPI	Derwent World Patents Index
IPC	International Patent Classification
MERS	Middle East Respiratory Syndrome
BSL-4	Biosafety Level 4
WHO	World Health Organization
SARS-CoV-2	Severe Acute Respiratory Syndrome of Coronavirus-2
BSC	Biosafety Cabinet
HCBL	High-Containment Biological Laboratory
CNAS	China National Accreditation Service
GMO	Genetically Modified Organism
USA	United States
PAPR	Powered Air-Purifying Respirator
BSH	Biosafety Hood
R&D	Research and Development
LBM	Laboratory Biosafety Manual
