# Supplementary material for: Global Patent Landscape and Technological Trends in Biosafety Level 3 (BSL-3) Laboratories Technologies
Source: BioTech (Basel). 2026 Jul 10;15(3):52. doi: 10.3390/biotech15030052 (PMC13397928; doi:10.3390/biotech15030052)
Supplement: Supplementary file 1 [file biotech-15-00052-s001.zip › biotech-4387600 - Supplementary Table S1.pdf]

Supplementary Materials: Global Patent Landscape and Technological Trends in Biosafety Level 3 (BSL-3) Laboratories Technologies

Milca de J. Silva, Roni D. Vinhas, Helena S. da Hora, Saada L. C. Fernandez, Hayna Malta-Santos, Hugo Saba, Camila D. F. Ribeiro, Marilda de S. Gonçalves and Bruna A. S. Machado

Table S1. Top patent applicants in BSL-3 technologies, showing leading institutions and companies, with their respective percentage share of total filings.

Nº	Applicant	Percentage (%)
1	Wuhan Institute of Virology CAS	5,17
2	Chengdu Bus Co. Ltd.	3,45
3	Shanghai Jiehao Biotechnology Co. Ltd.	3,45
4	Sanitary Equipment Institute Academy of Military Medical Sciences PLA	1,72
5	Shanghai Radobio Scientific Co. Ltd.	1,72
6	Zhuhai Guangtong Auto Co. Ltd.	1,72
7	CooperSurgical Inc.	1,72
8	Nanjing Bosen Technology Co. Ltd.	1,72
9	Shenzhen Xige Industrial Co. Ltd.	1,72
10	Chongqing Orientaldata Technology Co. Ltd.	1,72
11	Hainan Viewkr Bio-tech. Co. Ltd.	1,72
12	Dream Lab Technology (Shanghai) Co. Ltd.	1,72
13	The 8th Medical Center of Chinese PLA General Hospital	1,72
14	Ippr Laboratory System Technology (Beijing) Co. Ltd.	1,72
15	ADILSON SILVA JUNIOR	1,72
16	Chongqing Bluehorizon Energy Saving Technology Co. Ltd.	1,72
17	Harbin Veterinary Research Institute CAAS	1,72
18	Shaanxi Meili-OH Animal Health Co. Ltd.	1,72
19	Shanghai Hushi Laboratory Equipment Co. Ltd.	1,72
20	Zhongke Meiling Cryogenics Co. Ltd.	1,72
21	Wuxi Lamoton Technology Co. Ltd.	1,72
22	Tianjin Hanaco Medical Co. Ltd.	1,72
23	Shenzhen Wanwei Air Conditioning Purification Engineering Co. Ltd.	1,72
24	Guangzhou Lanjing Environmental Protection Technology Development Co. Ltd.	1,72
25	Hainan Chuanyi Industrial Co. Ltd.	1,72
26	Military Veterinary Research Institute Academy of Military Medical Sciences	1,72
27	Suzhou Huatuo Biotechnology Co. Ltd.	1,72
28	Shanghai Etopia Building Technology Co. Ltd.	1,72
29	University of Washington	1,72
30	Tongji University	1,72
31	XOMA (US) LLC	1,72
32	China Electronics System Engineering No.2 Construction Co. Ltd.	1,72
33	Becton Dickinson and Company	1,72
34	Gehring Harald	1,72
35	CAO Ru-an	1,72
36	GAO Hai	1,72
37	American Sterilizer Company	1,72
38	Beijing Anyutong Environmental Engineering And Technology Co. Ltd	1,72
39	Institute of Medical Biology Chinese Academy of Medical Sciences	1,72
40	BECARV SA	1,72

41	Guangxi Bossco Environmental Protection Technology Co. Ltd.	1,72
42	Jiangsu Kulinan Laboratory Equipment Co. Ltd.	1,72
43	Beijing Cleanair Biological Laboratory Engineering Co. Ltd.	1,72
44	Suzhou Purification Engineering Installation Co. Ltd.	1,72
45	Zhenjiang Kangfei Automobile Manufacturing Co. Ltd.	1,72
46	Changsha Xieda Biological Technology Co. Ltd.	1,72
47	Nanjing Chuanye Environmental Protection Technology Co. Ltd.	1,72
48	China Academy of Building Research Co. Ltd.	1,72
49	Shandong Bsd Environmental Protection Technology Co. Ltd.	1,72
50	Shandong Aokunlai Intelligent Technology Co. Ltd.	1,72
51	Beijing Xinji Yongkang Biological Technology Co. Ltd.	1,72
52	Tianjin Changte Purification Technology Co. Ltd.	1,72
53	SHEN Zhao-long	1,72
