# Supplementary material for: Global Patent Landscape and Technological Trends in Biosafety Level 3 (BSL-3) Laboratories Technologies
Source: BioTech (Basel). 2026 Jul 10;15(3):52. doi: 10.3390/biotech15030052 (PMC13397928; doi:10.3390/biotech15030052)
Supplement: Supplementary file 1 [file biotech-15-00052-s001.zip › biotech-4387600 - Supplementary Table S2.pdf]

Supplementary Materials: Global Patent Landscape and Technological Trends in Biosafety Level 3 (BSL-3) Laboratories Technologies

Milca de J. Silva, Roni D. Vinhas, Helena S. da Hora, Saada L. C. Fernandez, Hayna Malta-Santos, Hugo Saba, Camila D. F. Ribeiro, Marilda de S. Gonçalves and Bruna A. S. Machado

Table S2: Description of patent documents related to BSL-3 technologies.

Publication No. / Date	Title	Technology	Country	IPC Class	Assignee	Ref
CN101711935A / 2009-10-19	High Efficiency Air Filter Unit	Ventilation: HEPA filtration unit	CN	B01	Lanzhou Veterinary Research Institute, Chinese Academy of Agricultural Sciences, Lanzhou, Gansu, China./Institute of Medical Equipment, Academy of Military Medical Sciences, Tianjin, China	1
US8137615B2 / 2011-01-14	Automated Workstation for Disinfecting Objects and Methods of Use Thereof	Equipment: Disinfection workstation	US	A61, A01	University of Washington, Seattle, WA, USA	2
US10294658B2 / 2011-07-13	Flexible Manufacturing System	Infrastructure: Modular manufacturing system	US	C02, B23, E04, G05	Xoma CorporationBerkeley, CA. USA.	3
CN103848462B / 2014-01-14	BSL-3 Laboratory Wastewater Centralized Processing System丨BSL-3	Waste Management: Centralized wastewater system	CN	C02, G01	China Electronics System Engineering No. 2 Construction Co., Ltd, Wuxi, Jiangsu, China	4
US9446159B2 / 2014-10-02	Flow Cytometer Biosafety Hood and Systems Including the Same	Analytical Equipment: Flow cytometer with biosafety hood	US	A61, B01, G01	Becton Dickinson and Company (BD), Franklin Lakes, NJ, USA	5
DE202015008363U1 / 2015-12-03	Depressurization Flash Tank with Water Trap or Intermediate Diaphragm for Safety Devices with Biosafety Level (BSL) 1-2-3 And 4 Regions	Containment Equipment: Pressure relief container	DE	B65, B01	Gehring Technologies GmbH, Ostfildern, Germany	6
CN105865870A / 2016-04-12	Specimen Pretreatment Equipment	Lab Equipment: Specimen pretreatment machine	CN	G01, B65	Cao R	7
CN106245944B / 2016-06-15	Analyzing And Detecting the Base Laboratory Building Module	Construction Module: Base lab unit with air purification	CN	E04	Gao H	8

IN404564B / 2016-06-20	Portable Decontamination Unit	Decontamination: Biosafety cabinet sterilization system	IN		American Sterilizer Company ǀSteris Corporation, OH, USA	9
CN205974094U / 2016-08-29	Laboratory Sewage Treatment Device	Waste Management: Sewage treatment box	CN	C02	Beijing Anyutong Environmental Engineering & Technology Co., Ltd., Beijing, China	10
CN206768091U / 2017-05-31	A Biological Safety Laboratory Experiment Operating Platform	Workstation: Biosafety experimental platform	CN	C12	Institute of Medical Biology Chinese Academy of Medical Sciences, Kunming, Yunnan, China	11
BE1025019B1 / 2017-07-28	Assembly Comprising a Framework and at Least One First Element to Be Connected	Infrastructure: Cleanroom ceiling assembly	BE	E04	Becarv Sa, Buenos Aires, Argentina	12
CN207877519U / 2017-12-29	Integrated Laboratory Sewage Treatment Device	Waste Management: Integrative sewage system	CN	C02	Guangxi Bossco Environmental ProtectionTechnology Co., Ltd., Nanning, Guangxi, China	13
CN108328817A / 2018-02-01	A Concentrated-Processing Laboratory Sewage Treatment Device	Waste Management: Centralized processing system	CN	C02	Jiangsu Kulinan Laboratory Equipment Co., Ltd, Taizhou, Jiangsu, China	14
CN208462175U / 2018-06-27	A Biological Safe Laboratory Full Automatic Comprehensive Control Cabinet	Control Systems: Automated control cabinet	CN	H05	Beijing Cleanair Biological Laboratory Engineering Co., Ltd., Beijing, China	15
CN210217221U / 2018-09-27	Integrated Laboratory System	Infrastructure: Integrated laboratory system	CN	E04, B01	Suzhou Purification EngineeringInstallation Co., Suzhou, Jiangsu, China	16
CN209261264U / 2018-11-28	A Shelter Combined BSL-3 Laboratory	Animal Facilities: BSL lab with IVC cage and exhaust filter	CN	E04, A01, F24	Zhenjiang Kangfei Automobile Manufacturing Co., Ltd., Zhenjiang, Jiangsu, China	17

CN209673388U / 2019-02-26	A Quantitative Sealing Excrement Special Collection Tube	Sampling Tools: Sealed excrement collection pipe	CN	G01	Changsha Xieda Biological Technology Co, Changsha, Hunan, China	18
CN210237599U / 2019-06-25	Pre-Vertical Fermentation Structure Applied to the Sewage Treatment System of Biosafety Laboratory	Waste Management: Fermentation structure for sewage treatment	CN	C12	Nanjing Chuanye Environmental Protection Technology Co., Ltd, Nanjing, Jiangsu, China	19
CN110216719B / 2019-07-05	A Biological Experiment Protective Device for Biological Safety Laboratory	Protection: Biosafety lab protective gear system	CN	B25, B01, B08	China Academy of Building Research Co Ltd.| North China Electric Power University, Beijing, China	20
CN210736423U / 2019-08-28	Integrated Laboratory Sewage Comprehensive Processing Device	Waste Management: Comprehensive sewage device	CN	C02	Shandong Bsd Environmental Protection Technology Co., Ltd., Jinan, Shandong, China	21
CN210656591U / 2019-08-30	Combined Laboratory Sewage Comprehensive Processing Device	Waste Management: Combined sewage treatment unit	CN	C02	Shandong Aokunlai Intelligent Technology Co., Ltd., Jinan, Shandong, China	22
CN211382920U / 2019-12-04	A Biological Safety Laboratory Fog Shower Room	Decontamination: Fog spraying chamber	CN	A61, F26	Shanghai Jiehao Biotechnology Co Ltd., Shanghai, China	23
CN211755061U / 2019-12-16	A Biological Safety Laboratory Flume	Infrastructure: Ferry groove with fumigation interface	CN	B01	Shanghai Jiehao Biotechnology Co Ltd, Shanghai, China	24
CN211612743U / 2019-12-26	A Biological Safety Cabinet	Workstation: Biosafety cabinet with waste barrel	CN	B01	Beijing Xinji Yongkang Biological Technology Co., Ltd., Beijing, China	25
CN211370144U / 2019-12-31	Airtight Door for Biological Safety Laboratory	Infrastructure: Airtight lab door with sealing ring	CN	E06	Academy of Military Sciences, Academy of System	26

					Engineering Medicine , Beijing, China丨Tianjin Changte Purification Engineering Co Ltd., Tianjin, China	
CN211508486U / 2020-03-17	A Special Device Belt for Biological Safety Laboratory	Cabling Infrastructure: Equipment belt with disinfection holes	CN	H02	Ippr Lab System Technology Co., Beijing, China	27
CN111502350A / 2020-03-27	Air Inflation Membrane Structure Virus Detection Laboratory, And Covering Structure Virus Detection Laboratory	Infrastructure: Inflatable virus detection lab	CN	E04, F24	Bgi Genomics Co Ltd., Shenzhen, Guangdong, China 丨Bgi Shenzhen Co Ltd., Shenzhen, Guangdong, China丨 University Tongji, Shanghai, China	28
CN116651536A / 2020-04-16	Biosafety Laboratory	Infrastructure: Inflatable structure for biosafety lab	CN	B01	Bgi Shenzhen Co Ltd., Shenzhen, Guangdong, China 丨Shanghai Etopia Building Technology Co., Ltd., Shanghai, China	29
JP03242451U / 2020-04-16	Inflatable Film Structure Virus Test Laboratory; Biosafety Test Laboratory; Topsoil Type Structure Virus Test Laboratory	Ventilation: Inflatable lab with heat exchange system	JP	E04	Bgi Genomics Co., Ltd., Shenzhen, Guangdong, China	30
BR202020014109U2 / 2020-07-09	Resistant Thermo-Resistant Tripsnizer Assembly with Threadable Hermetic Seal	Analytical Equipment: Trypsinizer for vaccine production	BR	B01	Adilson S	31
CN111913455B / 2020-08-13	An Intelligent Comprehensive Control System of Biological Safety Laboratory	Control Systems: Intelligent integrated monitoring system	CN	G05	Suzhou Huatuo Biotechnology Co., Ltd., Suzhou, Jiangsu, China	32

CN111957356A / 2020-08-13	Movable Biological Safety Laboratory	Infrastructure: Movable BSL lab with support and experiment cabins	CN	B01	Yinlong New Energy Co., Ltd., Zhuhai, Guangdong, China丨Zhuhai Guantong Automobile Co., Ltd., Zhuhai, Guangdong, China	33
US12005432B2 / 2020-10-08	Configurable Workstations	Workstation: Modular biological protocol station	US	G01, B01, C07, C12, G05, G06, G16, H04	Coopersurgical Inc., Trumbull, CT, USA	34
CN112140976A / 2020-10-23	Self-Propelled Biological Safety Laboratory	Infrastructure: Self-propelled BSL lab with HVAC	CN	B60	Chengdu Bus Co., Ltd., Chengdu, Sichuan, China	35
CN214083781U / 2020-10-23	Ventilation Pipeline System of Self-Propelled Biological Safety	Ventilation: Mobile BSL ventilation pipeline system	CN	B60	Chengdu Bus Co., Ltd., Chengdu, Sichuan, China	36
CN214620347U / 2021-03-03	Biological Safety Laboratory Sterilizing Device Convenient for Installation	Decontamination: Lab sterilizing box with UV	CN	F26, A61	Nanjing Bosen Technology Co., Ltd., Nanjing, Jiangsu, China	37
CN214581692U / 2021-03-15	An Air Sterilizing Device for Biological Safety	Air Sterilization: Biological safety air sterilizer	CN	F24	Shenzhen Xige Industry Co., Ltd., Shenzhen, Guangdong, China	38
CN113006542A / 2021-04-29	Biological Safety Secondary Laboratory Based on Negative Pressure Tent	Containment: Negative pressure tent lab	CN	E04, E06	Chongqing Oriental data Technology Co., Ltd. Chongqing, China	39
CN215314614U / 2021-06-30	A Biological Safety Cabinet	Workstation: Biosafety cabinet with sterilizing box	CN	B08, A61, B01	Hainan Viewkr Bio-Tech Co., Ltd., Haikou, Hainan, China	40
CN216223402U / 2021-11-12	Biological Safety Working Table with Hepa Filtering System and Ultraviolet Irradiation Device	Workstation: Bio-safety working table with HEPA and UV	CN	B01, A61	Dream Lab Technology (Shanghai) Co., Ltd., Shanghai, China	41

CN113914441A / 2021-11-25	Floor Drain for High Grade Biological Safety	Drainage: Lab floor drain for high-level biosafety	CN	E03, B02	Wuhan Virology Institute, Chinese Academy of Sciences, Wuhan, Hubei, China	42
CN114408414A / 2022-01-13	A Biological Safety Protecting Device for Biological Safety	Protection: Film-based safety device for labs	CN	B65	The Eighth Medical Center, Chinese PLA General Hospital (Beijing, China)	41
CN116115803A / 2022-09-07	A Multifunctional Safe Chemical Shower Disinfecting System and Method	Decontamination: Multifunctional sterilizing shower system	CN	A61	Wuhan Virology Institute, Chinese Academy of Sciences (CAS) (Wuhan, Hubei, China)	43
CN218530958U / 2022-10-14	Biological Safety Cabinet with Intelligent Exhaust Device	Workstation: Sealed biosafety cabinet with movable parts	CN	B01	Shanghai Radobio Science Co., Ltd., Shanghai, China	44
CN115466674A / 2022-10-27	Biological Sampling and Culturing Device for Biological Safety Laboratory	Culture Equipment: Biological sampling and cultivation system	CN	C12	Ippr Lab System Technology (Beijing) Co., Ltd., Beijing, China	45
CN218912384U / 2022-12-09	Full-Automatic Pcr Square Cabin Detection Laboratory	Infrastructure: Full-automatic PCR square cabin	CN	E04, C12	Chongqing Bluehorizon Energy Saving Technology, Co., Ltd. (Chongqing, China)	46
CN116105272A / 2022-12-20	Negative Pressure Control System of High-Grade Biological Safety Laboratory and Micro-Negative Pressure Sterilizing Method	Control Systems: Negative pressure control system	CN	F24	Harbin Veterinary Research Institute, Chinese Academy of Agricultural Sciences (CAAS) (Harbin, Heilongjiang, China)	47
CN219494298U / 2022-12-26	An Exhaust System for Biosafety Laboratory	Ventilation: Exhaust system with dual valves	CN	F24	Shaanxi Meili-Oh Animal Health Co., Ltd., Xi'an, Shaanxi, China	48

CN116065734A / 2022-12-28	Method For Setting Enclosure Structure of High-Grade Biological Safety Laboratory	Construction: Enclosure structure for BSL	CN	E04	Shanghai Hushi Laboratory Equipment Co., Ltd., Shanghai, China	49
CN116273215A / 2023-04-27	A Hepa Filter Box Body with Sealed Original Position and Using Method Thereof	Ventilation: HEPA filter box with sealed sterilization	CN	B01	Wuhan Virology Institute, Chinese Academy of Sciences (CAS), Wuhan, Hubei, China	50
CN221016102U / 2023-08-30	Conveyor Belt Type Bio-Safety Cabinet	Workstation: Conveyor belt biosafety cabinet	CN	B01, A61	Zhongke Meiling Cryogenics Co., Ltd., Hefei, Anhui, China	51
CN221122506U / 2023-11-13	Air Conditioning System and Tent Biological Safety Laboratory	Ventilation: Tent lab air conditioning with filtration	CN	F24	Wuxi Lamoton Technology Co., Ltd., Wuxi, Jiangsu, China	53
CN118066628A / 2024-01-23	Ventilated Air Filtration Sterilization Method and Related Apparatus	Ventilation: Cleanroom filtration and sterilization system	CN	F24	Tianjin Hanaco Medical Co., Ltd., Tianjin, China	54
CN118253560A / 2024-04-16	Air Exhausting and Purifying Device for Biosafety Laboratory	Ventilation: Exhaust air purification unit	CN	B08	Shenzhen Wanwei Air Conditioning Purific Co., Ltd., Shenzhen, Guangdong, China	55
CN222287342U / 2024-05-24	Biological Safety Cabinet with Stable Structure	Workstation: Biosafety cabinet with screen plate	CN	B01	Guangzhou Lanjing Environmental Technology Co., Ltd., Guangzhou, Guangdong, China	56
CN119330234A / 2024-11-01	Lifting Device for Biosafety Laboratory	Lifting Equipment: Lab hoisting device	CN	B66	Hainan Chuanyi Industry Co., Ltd., Haikou, Hainan, China.	57
CN221122506U / 2023-11-13	Air Conditioning System and Tent Biological Safety Laboratory	Ventilation: Tent lab air conditioning with filtration	CN	F24	Military Veterinary Research Institute Pla Academy of Military Sciences, Changchun, Jilin, China	53

References

1. High-efficiency air filtration unit []EB1.1. (2009).
2. Van Hooser, J. P. Automated Workstation For Disinfecting Objects And Methods Of Use Thereof. (2008).
3. Baarman, D. W. Miniature hydro-power generation system power management. (2009).
4. BSL-3 laboratory active-poison sewage centralized processing system. (2014).
5. US9446159B2 - Flow cytometer biosafety hood and systems including the same .
6. Espacenet – search results.
7. CN105865870A - Sample preprocessing apparatus .
8. Analysis detection basic equipment laboratory building module. (2016).
9. Derwent Innovation.
10. Portable decontamination system. (2006).
11. Derwent Innovation. https://www.derwentinnovation.com/tip-innovation/recordView.do?hideHighlightPanel=true&idType=uid/recordid&datasource=T3&databaseIds=PATENT&category=PAT&recordKeys=CN206768091U_20171219&TYPE=RECORDVIEW&fromExternalLink=true&fromLocation=external&isDAJImageAllowed=false. (accessed on 30 July 2025).12. BE1025019b1 - assembly comprising a framework and at least one first element to be connected | assemblage comprenant une ossature et au moins un premier element a relier.
13. Integrated laboratory sewage treatment device .
14. A concentrated-processing laboratory sewage treatment device . https://www.derwentinnovation.com/tip-innovation/recordView.do?hideHighlightPanel=true&idType=uid/recordid&datasource=T3&databaseIds=PATENT&category=PAT&recordKeys=CN108328817A_20180727&TYPE=RECORDVIEW&fromExternalLink=true&fromLocation=external&isDAJImageAllowed=false. (accessed on 28 July 2025).
15. Full -automatic composite control cabinet of biosafety laboratory.
16. Integrated laboratory system.
17. Patent Claim:. CN209261264U A modular container-based BSL-3 laboratory. (2018).
18. A quantitative sealing excrement special collection tube.
19. pre-vertical fermentation structure applied to the sewage treatment system of biosafety laboratory | Vertical fermentation structure for the pretreatment of laboratory wastewater.
20. A biological experiment protective device for biological safety laboratory.
21. Integrated laboratory sewage comprehensive treatment equipment. (2019).
22. Combined laboratory sewage comprehensive processing device | Modular Integrated Laboratory Wastewater Treatment Equipment. https://www.derwentinnovation.com/tip-innovation/recordView.do?hideHighlightPanel=true&idType=uid/recordid&datasource=T3&databaseIds=PATENT&category=PAT&recordKeys=CN210656591U_20200602&TYPE=RECORDVIEW&fromExternalLink=true&fromLocation=external&isDAJImageAllowed=false.
23. A biological safety laboratory fog shower room.
24. A biological safety laboratory flume | A type of biosafety laboratory transfer hatch..
25. A biological safety cabinet | A type of biosafety cabinet.
26. Airtight door for biological safety laboratory | 一. https://www.derwentinnovation.com/tip-innovation/recordView.do?hideHighlightPanel=true&idType=uid/recor-

did&datasource=T3&databaseIds=PATENT&category=PAT&recordKeys=CN211370144U_20200828&TYPE=RECORDVIEW&fromExternalLink=true&fromLocation=external&is-DAJImageAllowed=false (accessed on 30 July 2025).
27. A special device belt for biological safety laboratory .
28. Air inflation membrane structure virus detection laboratory, and covering structure virus detection laboratory | Inflatable membrane structure virus testing laboratory and earth-covered structure virus testing laboratory.
29. Biosafety laboratory |.
30. Inflatable film structure virus test laboratory; biosafety test laboratory; topsoil type structure virus test laboratory.
31. Resistant thermo-resistant tripsnizer assembly with threadable hermetic seal.
32. Intelligent integrated control system for a biosafety laboratory, comprises an equipment monitoring unit, a follow-up unit, an original position library, a comprehensive analysis unit, a personnel monitoring terminal, a processor.
33. Movable biological safety laboratory has technical support cabin and experiment cabin that are connected by water supply pipeline, and functional separation of technical support cabin and experimental cabin is realized.
34. Configurable workstations.
35. Self-propelled biological safety laboratory | A self-propelled biosafety laboratory.
36. Ventilation pipeline system of self-propelled biological safety.
37. Biological safety laboratory sterilizing device convenient for installation.
38. Air sterilizing device for biological safety, has middle portion of upper end of cabinet portion that is opened with air outlet hole and middle portion is horizontally installed with exhausting fan.
39. Biological safety secondary laboratory based on negative pressure tent.
40. A biological safety cabinet.
41. Bio-safety working table with heating, ventilation and air-conditioning filter system and ultraviolet irradiation device, has buckles whose two groups are matched and connected with two groups of clamping frames under normal condition.
42. Floor drain for high grade biological safety.
43. A multifunctional safe chemical shower disinfecting system and method.
44. Biological safety cabinet with intelligent exhaust device.
45. Biological sampling and culturing device for biological safety laboratory.
46. Full-automatic PCR square cabin detection laboratory.
47. Negative pressure control system of high grade biological safety laboratory and micro-negative pressure sterilizing method.
48. An exhaust system for biosafety laboratory.
49. Method for setting enclosure structure of high grade biological safety laboratory .
50. A HEPA filter box body with sealed original position and using method thereof.
51. Conveyor belt type bio-safety cabinet .
52. Biosafety negative pressure dissecting table.
53. Air conditioning system and tent biological safety laboratory (.
54. Ventilated air filtration sterilization method and related apparatus.

55. Exhaust purification device for biosafety laboratory.
56. Biological safety cabinet with stable structure .
57. Lifting device for biosafety laboratory .
